# Risk factors for coronary artery disease progression: A retrospective cohort study of clinical and biochemical predictors

**DOI:** 10.1097/MD.0000000000042706

**Published:** 2025-06-06

**Authors:** Peiyong Zhang, Xiaochen Liu, Nan Feng, Penghong Duan, Yonglong Guo, Caiyan Bai

**Affiliations:** a Cardiology Department, The First Affiliated Hospital of Xinxiang Medical University, Xinxiang, China.

**Keywords:** biomarkers, CAD progression, Coronary artery disease, fibrinogen, left ventricular ejection fraction, NT-proBNP, predictive modeling

## Abstract

This study aimed to identify clinical and biochemical predictors of coronary artery disease (CAD) progression in patients undergoing routine cardiovascular evaluation. A retrospective cohort study was conducted involving 183 patients with confirmed CAD (92 with progression and 91 without progression). Clinical data, echocardiographic parameters, and laboratory biomarkers were collected at baseline. CAD progression was assessed based on repeat coronary angiography after a follow-up period. Multivariate logistic regression was used to identify independent predictors of CAD progression. The predictive accuracy of the model was evaluated using the area under the receiver operating characteristic curve), and model fit was assessed using the Hosmer–Lemeshow test. Elevated interleukin-6 levels (OR = 3.051, 95% confidence interval: 1.164–7.999, *P* = .023) and triglycerides (approaching significance, odds ratio = 1.126, 95% confidence interval: 0.980–1.293, *P* = .093) were identified as key predictors of CAD progression. The final model showed good discrimination with an area under the receiver operating characteristic curve of 0.701 and an acceptable fit (Hosmer–Lemeshow test: *P* = .078). Elevated interleukin-6 and triglycerides levels are significant or near-significant predictors of CAD progression. These findings suggest that inflammatory and lipid biomarkers play critical roles in disease progression and may serve as valuable targets for therapeutic interventions in high-risk patients.

## 1. Introduction

Coronary artery disease (CAD) remains the leading cause of morbidity and mortality worldwide, despite advances in diagnostic and therapeutic strategies.^[1]^ CAD is characterized by the progressive narrowing and hardening of coronary arteries due to atherosclerosis, which can lead to myocardial infarction, heart failure, and sudden cardiac death.^[2]^ Identifying individuals at risk for CAD progression is crucial for preventing adverse cardiovascular events and improving patient outcomes.

Traditionally, the assessment of CAD has relied heavily on clinical evaluation, imaging techniques, and a limited set of biochemical markers.^[3]^ However, the complexity of CAD pathophysiology necessitates a more comprehensive approach that includes the identification of novel predictors that can provide insights into the underlying mechanisms driving disease progression. Biomarkers such as N-terminal pro-brain natriuretic peptide (NT-proBNP),^[4]^ C-reactive protein,^[5]^ and fibrinogen^[6]^ have emerged as potential indicators of cardiovascular risk, offering a window into the molecular processes that contribute to atherosclerosis and its complications.

Left ventricular ejection fraction (LVEF) is a well-established parameter that reflects the contractile function of the heart.^[7]^ It has been extensively used in the prognostication of various cardiovascular diseases, including CAD. Reduced LVEF is associated with adverse outcomes and has been proposed as a marker of disease severity and progression.^[8]^ Additionally, the role of lipid metabolism, particularly elevated cholesterol levels, in the pathogenesis of CAD is well-documented.^[9]^ Dyslipidemia accelerates the development of atherosclerotic plaques and promotes plaque instability, leading to increased cardiovascular risk.^[10]^

While several studies^[11–15]^ have investigated the prognostic significance of individual biomarkers and clinical parameters in CAD, there is a paucity of research that simultaneously examines multiple predictors to develop a comprehensive risk profile for CAD progression. Such an approach could enhance risk stratification and allow for more personalized therapeutic interventions. In this study, we aim to identify the clinical and biochemical predictors of CAD progression in a cohort of patients undergoing routine cardiovascular evaluation. We hypothesize that reduced LVEF, elevated biomarkers such as NT-proBNP and fibrinogen, and dyslipidemia are associated with an increased risk of CAD progression.

## 2. Materials and methods

### 2.1. Study design and setting

This was a retrospective cohort study conducted between January 2022 and January 2024 at the First Affiliated Hospital of Xinxiang Medical University, a tertiary care center specializing in cardiovascular diseases located in Xinxiang, China. The study aimed to identify clinical and biochemical predictors associated with the progression of CAD among patients undergoing routine cardiovascular evaluation and management. This research followed the STROBE guidelines.

Participants were recruited consecutively from patients attending the cardiology outpatient clinics and those admitted to the cardiology wards of Xinxiang Medical University. Eligibility was assessed based on medical records review and clinical evaluation by attending cardiologists. The study was conducted in accordance with the Declaration of Helsinki and was approved by the Institutional Review Board (IRB) of The First Affiliated Hospital of Xinxiang Medical University (Approval Number: 20220623). Written informed consent was obtained from all participants before enrollment. Participants were assured of the confidentiality of their information and their right to withdraw from the study at any time without any impact on their medical care.

### 2.2. Study population

#### 2.2.1. Inclusion criteria

- Adults aged ≥ 18 years diagnosed with CAD confirmed by coronary angiography.
- Patients with stable or unstable angina, or those who had experienced a myocardial infarction (MI) within the past 6 months.
- Individuals who provided written informed consent to participate in the study.

#### 2.2.2. Exclusion criteria

- Patients with a history of congenital heart disease, valvular heart disease, or cardiomyopathies other than ischemic cardiomyopathy.
- Individuals with chronic inflammatory diseases, autoimmune disorders, or active infectious diseases at the time of enrollment.
- Patients receiving immunosuppressive therapy or those with a history of malignancy within the past 5 years.
- Patients with uncontrolled hypertension (systolic blood pressure > 180 mm Hg or diastolic blood pressure > 110 mm Hg despite medical therapy).
- Patients with significant anemia (hemoglobin < 10 g/dL).
- Individuals with severe chronic obstructive pulmonary disease (FEV1 < 50% of predicted).
- Pregnant or lactating women.
- Individuals with renal insufficiency (eGFR < 30 mL/min/1.73 m²) or hepatic dysfunction (ALT/AST > 5 times upper limit of normal).

### 2.3. Sample size calculation

The sample size was calculated based on an anticipated difference in (primary outcome measure, e.g., biomarker levels) between patients with and without CAD progression. Assuming an effect size of (moderate risk; odds ratio of 2),^[11]^ a power of 80%, and a significance level (α) of 0.05, a minimum of 60 participants were required in each group. Accounting for potential dropouts and missing data, a total of 63 patients were enrolled, with 31 patients in the progression group and 32 patients in the non-progression group.

### 2.4. Data collection and measurements

#### 2.4.1. Clinical and demographic data

At baseline, comprehensive clinical and demographic data were collected through patient interviews and medical record reviews. The collected data included:

- Age and sex.
- Body mass index (BMI) calculated as weight in kilograms divided by height in meters squared.
- Lifestyle factors: smoking status (current, former, never), alcohol consumption (yes/no), and physical activity levels.
- Medical history: presence of hypertension, diabetes mellitus, dyslipidemia, family history of cardiovascular disease, and previous episodes of MI or stroke.
- Medication use: current use of beta-blockers, angiotensin-converting enzyme inhibitors, clopidogrel, warfarin, and other relevant medications.
- Vital signs: blood pressure, heart rate, and others as applicable.

#### 2.4.2. Echocardiographic assessment

All participants underwent comprehensive transthoracic echocardiography performed by experienced cardiologists using a standardized protocol and equipment (Philips EPIQ 7). The primary parameter measured was LVEF, which was assessed using the biplane Simpson method in accordance with the American Society of Echocardiography guidelines.^[16]^

#### 2.4.3. Laboratory evaluations

Fasting blood samples were collected from all participants after an overnight fast of at least 8 hours. Samples were processed and analyzed in the central laboratory following standardized procedures. The following biochemical parameters were measured:

- Lipid profile: total cholesterol, low-density lipoprotein cholesterol (LDL-C), high-density lipoprotein cholesterol, and triglycerides (TAG) using enzymatic colorimetric methods.
- Cardiac biomarkers: high-sensitivity troponin I/T levels measured using Elecsys Troponin T high-sensitive assays (Roche Diagnostics Asia Pacific Pte Ltd, China).
- Natriuretic peptides: brain natriuretic peptide (BNP) and NT-proBNP measured using ARCHITECT BNP assay (Abbott Laboratories) and Elecsys proBNP II assays (Roche Diagnostics Asia Pacific Pte Ltd, China), respectively.
- Glycemic control markers: fasting blood glucose and glycated hemoglobin levels measured using Tina-quant HbA1c Gen.3 (Roche Diagnostics Asia Pacific Pte Ltd, China).
- Inflammatory markers: high-sensitivity C-reactive protein measured by immunoturbidimetric assay, interleukin-6 (IL-6), and tumor necrosis factor-alpha measured by enzyme-linked immunosorbent assay (ELISA) using commercially available kits (DENARSE ELISA Kit, c-LEcta, China).
- Other biomarkers: homocysteine levels measured by Immulite 2000 Homocysteine (Siemens Healthineers), and fibrinogen levels measured by the Clauss method.
- All assays were performed in duplicate to ensure accuracy, and internal quality controls were maintained throughout the study period.

### 2.5. Definition and assessment of CAD progression

CAD progression was defined based on repeat coronary angiography performed after a follow-up period of 12 months or earlier if clinically indicated. Progression was characterized by:

- An increase in stenosis severity by ≥ 20% in any existing coronary artery lesion.
- Development of new significant stenosis (≥50% luminal narrowing) in previously unaffected coronary segments.
- Occurrence of adverse cardiac events such as MI, unstable angina requiring revascularization, or sudden cardiac death.

Coronary angiograms were analyzed by 2 independent interventional cardiologists blinded to the patients’ clinical and laboratory data to reduce observer bias. Discrepancies were resolved by consensus.

### 2.6. Statistical analysis

Data were entered into a secure, password-protected database using Microsoft Excel, with double-entry verification to minimize data entry errors. All data were anonymized to protect patient confidentiality. All statistical analyses were performed using STATA version 18.0 (StataCorp LLC, College Station, TX). Continuous variables were presented as means with standard deviations (SD) if they were normally distributed, or as medians with interquartile ranges for non-normally distributed data. The distribution of continuous variables was assessed using the Shapiro–Wilk test. Comparisons between patients with and without CAD progression were conducted using the Student *t* test for normally distributed variables or the Mann–Whitney *U* test for non-normally distributed variables. Categorical variables were expressed as frequencies and percentages and were compared using the Chi-square test or Fisher exact test, as appropriate.

To identify potential predictors of CAD progression, univariate logistic regression analyses were initially performed. Variables with a *P*-value of <.20 in univariate analyses were considered for inclusion in the multivariate logistic regression model. Multivariate logistic regression was conducted using a stepwise backward elimination approach to identify independent predictors of CAD progression while adjusting for potential confounders. The strength of associations was expressed as odds ratios (OR) with corresponding 95% confidence intervals (CI).

Multicollinearity among the variables was assessed using the variance inflation factor, and any variables with a variance inflation factor >10 were excluded from the final model. The goodness-of-fit for the logistic regression model was evaluated using the Hosmer–Lemeshow test, Akaike information criterion, and Bayesian information criterion. Additionally, the predictive accuracy of the final model was assessed by constructing a receiver operating characteristic curve and calculating the area under the curve. A two-tailed *P*-value of <.05 was considered statistically significant for all analyses.

## 3. Results

### 3.1. Clinicodemographic characteristics

A total of 183 patients with confirmed CAD were included in the study, with 91 patients exhibiting CAD progression and 92 without progression. There were no significant differences in baseline age, with a mean age of 63.46 years (SD, 5.12) in the non-progression group and 63.83 years (SD, 4.15) in the progression group (*P* = .598). Similarly, BMI showed no significant variation between the 2 groups, with a mean BMI of 25.11 kg/m² (SD, 2.73) in the non-progression group and 24.79 kg/m² (SD, 2.58) in the progression group (*P* = .425). The distribution of gender was also not significantly different, although there was a trend towards a higher proportion of females in the CAD progression group (52.75% vs 42.39%; *P* = .161).

No significant differences were observed for other cardiovascular risk factors, including smoking history (*P* = .186), alcohol intake (*P* = .924), family history of cardiovascular disease (*P* = .258), hypertension (*P* = .406), diabetes mellitus (*P* = .598), or myocardial infarction (*P* = .807). Medications such as beta-blockers and angiotensin-converting enzyme inhibitors were similarly distributed across groups. Notably, a lower proportion of patients with CAD progression were receiving warfarin (16.48% vs 32.61%; *P* = .011) (Table 1).

**Table 1:** Clinicodemographic characteristics of patients with CAD stratified by progression status.

	No CAD progression	CAD progression	*P*-value
	(N = 92)	(N = 91)	
Age (years)			
Mean (SD)	63.46 (5.12)	63.83 (4.15)	.598
Distribution – no. (%)			.926
<65 years	56 (60.86%)	56 (61.54%)	
≥65 years	36 (39.14%)	35 (38.46%)	
Body mass index			
Mean (SD)	25.11 (2.73)	24.79 (2.58)	.425
Distribution – no. (%)			.454
Underweight (<18.5 kg/m^2^)	42 (45.65%)	51 (56.04%)	
Normal (18.5–24.9 kg/m^2^)	1 (1.09%)	2 (2.19%)	
Overweight (25–29.9 kg/m^2^)	46 (50%)	36 (39.56%)	
Obese (30 kg/m^2^ or above)	3 (3.26%)	2 (2.19%)	
Gender – no. (%)			.161
Female	39 (42.39%)	48 (52.75%)	
Male	53 (57.61%)	43 (47.25%)	
Smoking history – no. (%)			.186
No	67 (72.83%)	58 (63.74%)	
Yes	25 (27.17%)	33 (36.26%)	
Alcohol intake – no. (%)			.924
No	57 (61.96%)	57 (62.64%)	
Yes	35 (38.04%)	34 (37.36%)	
Family history of cardiovascular disease – no. (%)			.258
No	76 (82.61%)	69 (75.82%)	
Yes	16 (17.39%)	22 (24.18%)	
Family history of hypertension – no. (%)			.406
No	68 (73.91%)	72 (79.12%)	
Yes	24 (26.09%)	19 (20.88%)	
Family history of diabetes mellitus – no. (%)			.598
No	73 (79.35%)	75 (82.42%)	
Yes	19 (20.65%)	16 (17.58%)	
Family history of myocardial infraction – no. (%)			.807
No	80 (86.96%)	78 (85.71%)	
Yes	12 (13.04%)	13 (14.29%)	
Dyslipidemia – no. (%)			.443
No	75 (81.52%)	70 (76.92%)	
Yes	17 (19.48%)	21 (23.08%)	
LVEF (%)			
Mean (SD)	46.85 (5.81)	47.51 (6.19)	.46
Distribution – no. (%)			.33
<40%	84 (91.30%)	79 (86.81%)	
>40%	8 (8.70%)	12 (13.19%)	
B-blocker use – no. (%)			.818
No	77 (83.69%)	75 (82.42%)	
Yes	15 (16.31%)	16 (17.58%)	
Clopidogrel use – no. (%)			.063
No	78 (84.78%)	67 (73.63%)	
Yes	14 (15.22%)	24 (26.37%)	
ACE-I use – no. (%)			.479
No	72 (78.26%)	75 (82.42%)	
Yes	20 (21.74%)	16 (17.58%)	
Warfarin use – no. (%)			.011
No	62 (67.39%)	76 (83.52%)	
Yes	30 (32.61%)	15 (16.48%)	

ACE-I = angiotensin-converting enzyme inhibitor, BMI = body mass index, CAD = coronary artery disease, CVD = cardiovascular disease, LVEF = left ventricular ejection fraction, MI = myocardial infarction, no. = number of patients in each group, SD = standard deviation.

### 3.2. Laboratory data and biomarkers

Patients with CAD progression had elevated levels of several key biomarkers. The mean TAG levels were significantly higher in the progression group (3.97 mmol/L, SD 2.77) compared to the non-progression group (3.17 mmol/L, SD 2.04; *P* = .027). Additionally, IL-6 levels were more frequently elevated in the progression group, with 18.68% of patients showing elevated IL-6 (>5 pg/mL), compared to only 8.70% in the non-progression group (*P* = .049).

Although other markers such as total cholesterol, LDL-C, and high-density lipoprotein cholesterol were elevated in both groups, the differences were not statistically significant (*P* > .05 for all). Troponin levels showed a trend towards elevation in the progression group (mean 0.023 µg/L, SD 0.009 vs 0.021 µg/L, SD 0.009; *P* = .094), while BNP and NT-proBNP were also elevated but did not reach statistical significance (*P* = .404 and *P* = .054, respectively) (Table 2).

**Table 2:** Laboratory data and biomarkers of patients with CAD stratified by progression status.

	No CAD progression	CAD progression	*P*-value
	(N = 92)	(N = 91)	
Total cholesterol			
Mean (SD)	6.49 (3.13)	7.08 (2.99)	.194
Distribution – no. (%)			.261
Normal (<5.2 mmol/L)	33 (35.87%)	23 (25.27%)	
Elevated (5.2–6.1 mmol/L)	10 (10.87%)	14 (15.38%)	
High risk (≥6.2 mmol/L)	49 (53.26%)	54 (59.34%)	
LDL-C			
Mean (SD)	4.21 (2.92)	3.81 (2.42)	.328
Distribution – no. (%)			.829
Normal (<3.4 mmol/L)	48 (52.17%)	48 (52.75%)	
Elevated (3.4–4.0 mmol/L)	1 (1.09%)	2 (2.19%)	
High risk (≥4.1 mmol/L)	43 (46.74%)	41 (45.05%)	
HDL-C			
Mean (SD)	2.32 (1.84)	2.5 (1.8)	.522
Distribution – no. (%)			.327
Normal	43 (46.74%)	36 (39.56%)	
Elevated (<1.0 for men and 1.3 mmol/L for women)	49 (53.26%)	55 (60.44%)	
TAG			
Mean (SD)	3.17 (2.04)	3.97 (2.77)	.027
Distribution – no. (%)			.246
Normal (<1.7 mmol/L)	26 (28.26%)	19 (20.88%)	
Elevated (≥1.7 mmol/L)	66 (71.74%)	72 (79.12%)	
Troponin			
Mean (SD)	0.021 (0.009)	0.023 (0.009)	.094
Distribution – no. (%)			.133
Normal (<0.01 μg/L)	27 (29.35%)	18 (19.78%)	
Elevated (>0.04 μg/L)	65 (70.65%)	73 (80.22%)	
BNP			
Mean (SD)	74.66 (46.95)	81.32 (60.10)	.404
Distribution – no. (%)			.052
Normal (<100 pg/mL)	91 (98.91%)	85 (93.41%)	
Elevated (>100 pg/mL)	1 (1.09%)	6 (6.59%)	
NT-proBNP			
Mean (SD)	99.85 (18.93)	116.53 (80.62)	.054
Distribution – no. (%)			.13
Normal	86 (93.48%)	79 (86.81%)	
Elevated (>125 pg/mL < 75 years and > 450 pg/mL > 75 years)	6 (6.52%)	12 (13.19%)	
HbA1C			
Mean (SD)	7.4 (0.65)	7.39 (0.53)	.97
Distribution – no. (%)			.711
Normal (<5.7%)	0 (0%)	0 (0%)	
Prediabetic (5.7–6.4%)	4 (4.35%)	3 (3.30%)	
Diabetic (≥6.5%)	88 (95.65%)	88 (96.70%)	
CRP			
Mean (SD)	11.88 (1.61)	11.8 (1.68)	.742
Distribution – no. (%)			–
Normal (<3 mg/L)	0 (0%)	0 (0%)	
High risk (>3 mg/L)	92 (100%)	91 (100%)	
IL-6			
Mean (SD)	3.98 (0.81)	3.99 (0.92)	.946
Distribution – no. (%)			.049
Normal (<5 pg/mL)	84 (91.30%)	74 (81.32%)	
Elevated (>5 pg/mL)	8 (8.70%)	17 (18.68%)	
TNF-alpha			
Mean (SD)	1.11 (0.55)	1.11 (0.49)	.975
Distribution – no. (%)			–
Normal (0–8.1 pg/mL)	92 (100%)	91 (100%)	
Elevated (>8.1 pg/mL)	0 (0%)	0 (0%)	
FBG			
Mean (SD)	8.25 (2.08)	8.44 (2.02)	.533
Distribution – no. (%)			.435
Normal (<5.6 mmol/L)	11 (11.96%)	9 (9.89%)	
Prediabetic (5.6–6.9 mmol/L)	11 (11.96%)	17 (18.68%)	
Diabetic (≥7.0 mmol/L)	70 (76.09%)	65 (71.42%)	
Homocysteine			
Mean (SD)	6.86 (1.83)	6.83 (1.88)	.931
Distribution – no. (%)			–
Normal (5–15 Mmol/L)	92 (100%)	91 (100%)	
Elevated (16–100 Mmol/L)	0 (0%)	0 (0%)	
Fibrinogen			
Mean (SD)	3.25 (1.08)	3.24 (1.12)	.933
Distribution – no. (%)			.967
Normal (<4 g/L)	70 (76.09%)	69 (75.82%)	
Elevated (>4 g/L)	22 (23.91%)	22 (24.16%)	

BNP = brain natriuretic peptide, CAD = coronary artery disease, CRP = C-reactive protein, HDL = high-density lipoprotein, IL = interleukin, LDL-C = low-density lipoprotein C, no. = number of patients in each group, NT-proBNP = N-terminal fragment of pro-b-type natriuretic peptide, SD = standard deviation, TNF = tumor necrotic factor.

### 3.3. Univariate logistic regression analysis

Univariate analysis identified elevated TAG as a significant predictor of CAD progression (OR = 1.158, 95% CI: 1.013–1.323, *P* = .032). Other variables, such as troponin (OR = 5.09E + 11, 95% CI: 0.0087–2.96E + 25, *P* = .096) and BNP (OR = 6.424, 95% CI: 0.758–54.463, *P* = .088), approached statistical significance but did not meet the threshold for inclusion (*P* < .05). IL-6 was identified as a near-significant factor (OR = 2.412, 95% CI: 0.984–5.913, *P* = .054) (**Table 3**).

**Table 3:** Unadjusted univariate logistic regression analysis of risk factors of CAD progression.

	OR	SE	Z	*P*-value	Low CI	High CI
Age (per year)	1.017	0.033	0.530	.596	0.955	1.083
BMI (per unit)	0.956	0.054	-0.800	.424	0.856	1.067
LVEF < 40% [Reference: >40%]	1.595	0.770	0.970	.333	0.619	4.107
Serum cholesterol (per unit)	1.065	0.052	1.300	.194	0.968	1.172
LDL-C (per unit)	0.947	0.053	-0.980	.327	0.849	1.056
HDL-C (per unit)	1.054	0.086	0.640	.521	0.898	1.237
TAG (per unit)	1.158	0.079	2.140	.032	1.013	1.323
Troponin (per unit)	5.09E + 11	8.23E + 12	1.67	.096	0.0087	2.96E + 25
BNP (>100 pg/mL) [Reference: <100 pg/mL]	6.424	7.006	1.710	.088	0.758	54.463
NT-proBNP (per unit)	1.008	0.005	1.580	.114	0.998	1.017
HbA1C (per unit)	0.991	0.246	-0.040	.970	0.609	1.611
CRP (per unit)	0.971	0.087	-0.330	.741	0.814	1.158
IL-6 (>5 pg/mL) [Reference: <5 pg/mL]	2.412	1.103	1.920	.054	0.984	5.913
TNF-alpha (per unit)	1.009	0.285	0.030	.976	0.580	1.754
FBG (per unit)	1.046	0.076	0.630	.531	0.908	1.206
Homocysteine (per unit)	0.993	0.080	-0.090	.931	0.849	1.162
Fibrinogen (per unit)	0.989	0.133	-0.080	.933	0.760	1.286
Male gender [Reference: Female]	0.659	0.196	-1.400	.161	0.368	1.181
Smoking [Reference: No]	1.525	0.488	1.320	.188	0.814	2.856
Alcohol intake [Reference: No]	0.971	0.296	-0.100	.924	0.534	1.766
History of Cardiovascular disease [Reference: No]	1.514	0.558	1.130	.260	0.736	3.117
History of hypertension [Reference: No]	0.748	0.262	-0.830	.407	0.376	1.486
History of DM [Reference: No]	0.820	0.309	-0.530	.598	0.391	1.716
Dyslipidemia [Reference: No]	1.324	0.485	0.770	.444	0.646	2.713
History of MI [Reference: No]	1.111	0.479	0.240	.807	0.478	2.585
B-blocker use [Reference: No]	1.095	0.432	0.230	.818	0.506	2.372
Clopidogrel use [Reference: No]	1.996	0.749	1.840	.066	0.956	4.164
ACE-I use [Reference: No]	0.768	0.287	-0.710	.480	0.369	1.598
Warfarin use [Reference: No]	0.408	0.147	-2.490	.013	0.202	0.825

ACEI = angiotensin-converting enzyme inhibitor, BNP = brain natriuretic peptide, CAD = coronary artery disease, CI = confidence interval, CRP = C-reactive protein, CVD = cardiovascular disease, HDL = high-density lipoprotein, IL-6 = interleukin-6, LVEF = left ventricular ejection fraction, MI = myocardial infarction, NT-proBNP = N-terminal fragment of pro-b-type natriuretic peptide, OR = odds ratio, P = *P*-value, SE = standard error, TAG = triglyceride, TNF = tumor necrotic factor.

### 3.4. Multivariable logistic regression analysis

In the multivariate analysis, elevated IL-6 levels (>5 pg/mL) were identified as an independent predictor of CAD progression, with patients exhibiting elevated IL-6 having significantly higher odds of disease progression (adjusted odds ratio [OR] = 3.051, 95% CI: 1.164–7.999, *P* = .023). TAG also approached significance as an independent predictor (adjusted OR = 1.126, 95% CI: 0.980–1.293, *P* = .093). Other biomarkers, such as troponin, BNP, and NT-proBNP, while elevated in the progression group, did not reach statistical significance in the multivariate model (*P* > .05 for all) (Table 4).

**Table 4:** Adjusted multivariable logistic regression analysis of risk factors of CAD progression.

	Multivariable Logistic Regression Model						Model Fit Statistics			
	aOR	SE	Z	*P*-value	Low CI	High CI	AUC	AIC	BIC	Hosmer–Lemeshow chi^2^ (df)/*P*
Male gender	0.858	0.282	-0.460	.642	0.451	1.635	0.552	255.71	262.13	–/.00
Smoking history	1.713	0.608	1.520	.129	0.855	3.433	0.545	255.93	262.35	–/.00
Clopidogrel use	1.644	0.672	1.220	.224	0.738	3.661	0.555	254.19	260.61	–/.00
Warfarin use	0.511	0.200	-1.720	.086	0.238	1.100	0.581	251.17	257.59	–/.00
Total cholesterol (per unit increase)	1.067	0.058	1.200	.232	0.959	1.188	0.552	255.98	262.4	–/.00
TAG (per unit increase)	1.126	0.080	1.680	.093	0.980	1.293	0.589	252.56	258.98	–/.00
Troponin (per unit increase)	2.59E + 08	4.50E + 09	1.11	.265	4.16E-07	1.61E + 23	0.573	254.84	261.27	–/.00
BNP (>100 pg/mL)	2.786	3.518	0.810	.417	0.234	33.101	0.527	253.52	259.94	–/.00
NT-proBNP (per unit increase)	1.005	0.006	0.870	.382	0.994	1.016	0.521	253.16	259.58	–/.00
IL-6 (>5 pg/mL)	3.051	1.500	2.270	.023	1.164	7.999	0.549	253.74	260.16	–/.00
Final model	–	–	–	–	–	–	0.701	248.830	284.130	14.14 (8)/.078

AIC = Akaike information criterion, aOR = adjusted odds ratio, AUC = ROC curve – area under the curve, BIC = Bayesian information criterion, BNP = brain natriuretic peptide, CAD = coronary artery disease, CI = confidence interval, CRP = C-reactive protein, IL-6 = interleukin-6, NT-proBNP = N-terminal fragment of pro-b-type natriuretic peptide, SE = standard error, TAG = triglyceride.

### 3.5. Model performance and fit

The final logistic regression model demonstrated good discrimination, with an area under the curve of 0.701. The model’s goodness-of-fit was assessed using the Hosmer–Lemeshow test, yielding a chi-squared value of 14.14 (8 degrees of freedom), with a non-significant *P*-value of .078, indicating that the model fits the data well (Table 4).

## 4. Discussion

This study aimed to identify clinical and biochemical predictors of CAD progression in a cohort of patients undergoing routine cardiovascular evaluation. By expanding the sample size and incorporating additional biomarkers into multivariate models, we found that elevated IL-6 levels and TAG (marginally significant) were important risk factors for CAD progression. These findings enhance our understanding of CAD pathophysiology and underscore the role of inflammatory markers and lipid metabolism in disease progression.

### 4.1. Elevated IL-6 as a key predictor

Among the predictors of CAD progression, IL-6 emerged as a significant independent risk factor. Elevated IL-6 levels (>5 pg/mL) were strongly associated with CAD progression, as reflected by the nearly 3-fold increased risk in patients with elevated levels (adjusted OR = 3.051, *P* = .023). This finding aligns with previous research linking chronic inflammation to the pathogenesis and progression of atherosclerosis.^[17–19]^ IL-6 is a pro-inflammatory cytokine that plays a central role in the inflammatory response and has been implicated in the destabilization of atherosclerotic plaques, leading to adverse cardiovascular events.^[20–22]^ Our results further reinforce the notion that IL-6 is not only a marker of systemic inflammation but also an active participant in the progression of coronary artery lesions.

In line with this, several recent studies have suggested that targeting IL-6 and other inflammatory pathways could represent a novel therapeutic approach in high-risk CAD patients.^[21,23,24]^ The Canakinumab Anti-Inflammatory Thrombosis Outcomes Study (CANTOS), for example, demonstrated that IL-1β inhibition significantly reduced recurrent cardiovascular events by attenuating downstream IL-6 signaling.^[25,26]^ As such, our findings suggest that IL-6 could serve as a valuable biomarker for risk stratification and could be integrated into clinical decision-making to identify patients who may benefit from anti-inflammatory therapies.

### 4.2. Lipid metabolism and TAGs

In addition to IL-6, elevated TAG levels were associated with a higher likelihood of CAD progression, approaching statistical significance in the multivariable model (adjusted OR = 1.126, *P* = .093). Elevated TAG levels are well-established risk factors for cardiovascular disease, and their role in atherogenesis is increasingly recognized. The atherogenic potential of TAG-rich lipoproteins, particularly their contribution to endothelial dysfunction and plaque formation, has been widely documented.^[27,28]^ Moreover, TAGs contribute to residual cardiovascular risk even after LDL-C levels are optimized through statin therapy.^[29]^

While total cholesterol and LDL-C did not show significant associations with CAD progression in our cohort, likely due to effective lipid-lowering therapy, the trend toward elevated TAGs underscores the need for comprehensive lipid management strategies. Emerging therapies, such as icosapent ethyl and fibrates, may offer additional benefit in patients with persistently elevated TAGs despite statin therapy.^[30]^

### 4.3. Biomarkers and cardiac function

Interestingly, traditional cardiac biomarkers, including NT-proBNP, troponin, and BNP, while elevated in patients with CAD progression, did not reach statistical significance in the multivariate analysis. NT-proBNP has been widely used as a prognostic marker in heart failure and CAD, reflecting myocardial stress and neurohormonal activation.^[31]^ The lack of statistical significance in our study could be attributed to the relatively low levels of NT-proBNP and BNP observed in our cohort, with most patients maintaining levels within normal or near-normal ranges.

Additionally, LVEF was not found to be a significant predictor of CAD progression, consistent with previous studies suggesting that LVEF is more closely related to heart failure outcomes than to atherosclerotic disease progression.^[32,33]^ Nonetheless, monitoring cardiac function remains essential in the overall management of CAD patients, particularly those at risk for heart failure.

### 4.4. Implications for clinical practice

Our findings highlight the importance of incorporating novel biomarkers, particularly IL-6, into the risk stratification of CAD patients. The addition of inflammatory markers such as IL-6 to traditional risk factors could improve the identification of patients at high risk of disease progression, allowing for more tailored therapeutic interventions. As demonstrated by our study, IL-6 was a stronger predictor of CAD progression than many traditional cardiovascular risk factors, further supporting its clinical utility.

Moreover, the association between TAGs and CAD progression underscores the need for aggressive lipid management, especially in patients with elevated TAG levels. Future guidelines should consider the inclusion of TAG-lowering therapies in patients with high residual cardiovascular risk, as well as the use of anti-inflammatory agents in those with elevated IL-6 levels.

### 4.5. Study limitations

Despite the strengths of our study, including the expanded sample size and comprehensive biomarker assessment, several limitations must be acknowledged. First, the study was conducted in a single center, which may limit the generalizability of our findings to broader populations. Second, the retrospective nature of the analysis precludes causal inference, and prospective studies are needed to confirm our results. Finally, while IL-6 and TAGs were identified as significant predictors, further research is warranted to explore the mechanisms underlying their role in CAD progression and to assess their potential as therapeutic targets.

In conclusion, this study identifies elevated IL-6 and TAGs as key predictors of CAD progression, providing new insights into the inflammatory and metabolic pathways driving disease advancement. These findings emphasize the importance of integrating novel biomarkers into clinical practice for improved risk stratification and personalized treatment strategies. Future research should focus on the therapeutic implications of targeting IL-6 and TAG-rich lipoproteins in patients at high risk of CAD progression.

## Author contributions

**Conceptualization:** Peiyong Zhang, Caiyan Bai.

**Data curation:** Xiaochen Liu, Nan Feng, Penghong Duan, Yonglong Guo.

**Formal analysis:** Peiyong Zhang, Xiaochen Liu.

**Investigation:** Xiaochen Liu, Nan Feng, Penghong Duan, Yonglong Guo.

**Methodology:** Xiaochen Liu, Nan Feng, Penghong Duan, Yonglong Guo.

**Project administration:** Caiyan Bai.

**Resources:** Caiyan Bai.

**Software:** Caiyan Bai.

**Supervision:** Peiyong Zhang, Caiyan Bai.

**Validation:** Peiyong Zhang, Caiyan Bai.

**Visualization:** Peiyong Zhang, Xiaochen Liu.

**Writing – original draft:** Peiyong Zhang, Xiaochen Liu.

**Writing – review & editing:** Nan Feng, Penghong Duan, Yonglong Guo, Caiyan Bai.
